# Supplementary material for: Quantifying 3′UTR length from scRNA-seq data reveals changes independent of gene expression
Source: Nat Commun. 2024 May 14;15:4050. doi: 10.1038/s41467-024-48254-9 (PMC11094166; doi:10.1038/s41467-024-48254-9)
Supplement: Supplementary file 1 — Supplementary Information [file 41467_2024_48254_MOESM1_ESM.pdf]

Fansler, Mitschka & Mayr, Supplementary Figure 1

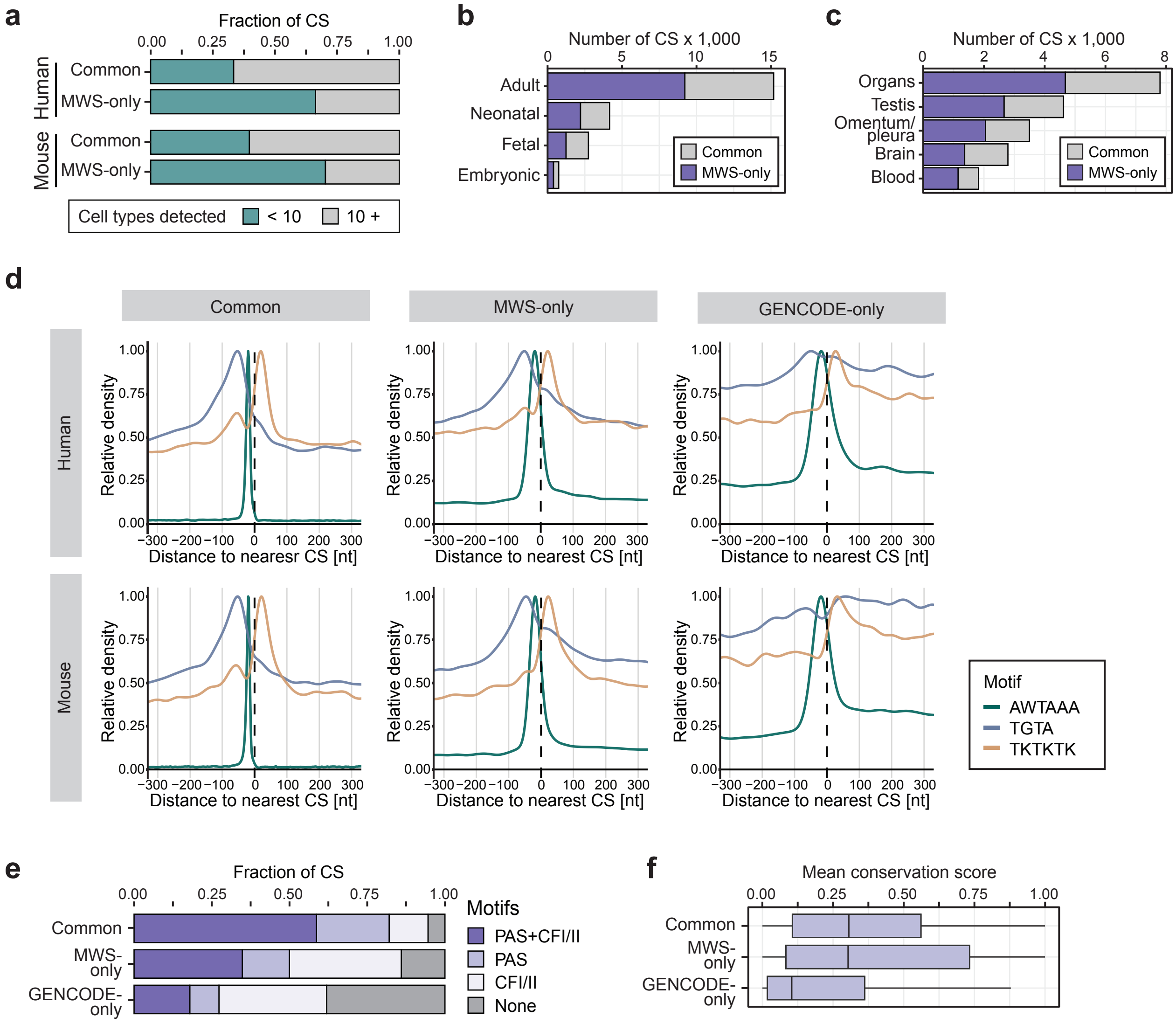

Supplementary Figure 1. Characterization of mRNA 3′ end CS in 206 primary cell types.
a Fraction of common and MWS-only CS that were detected in fewer than ten cell types. Shown are CS from human and mouse MWS annotations. **b** For cell type-restricted mouse CS (detected in < 10 cell types), the developmental stage of the cell types is shown. **c** As in (b), but the tissue of origin is shown. **d** Metaplots for binding site enrichment of CPA factors centered on the CS, including canonical PAS (AWTAAA, dark green), CFI (TGTA, blue) and CFII (TKTKTK, orange). **e** Motif distribution surrounding mouse CS (position 0). PAS (AWTAAA) in [-50,0], CFI binding site (TGTA) in [-100,0], CFII binding site (TKTKTK) in [0,50]. **f** Mean PhastCons score of 60 genomes in a 100-nt window centered on CS, but excluding coding sequences. Box shows IQR with median and whiskers 1.5*IQR.

Fansler, Mitschka & Mayr, Supplementary Figure 2

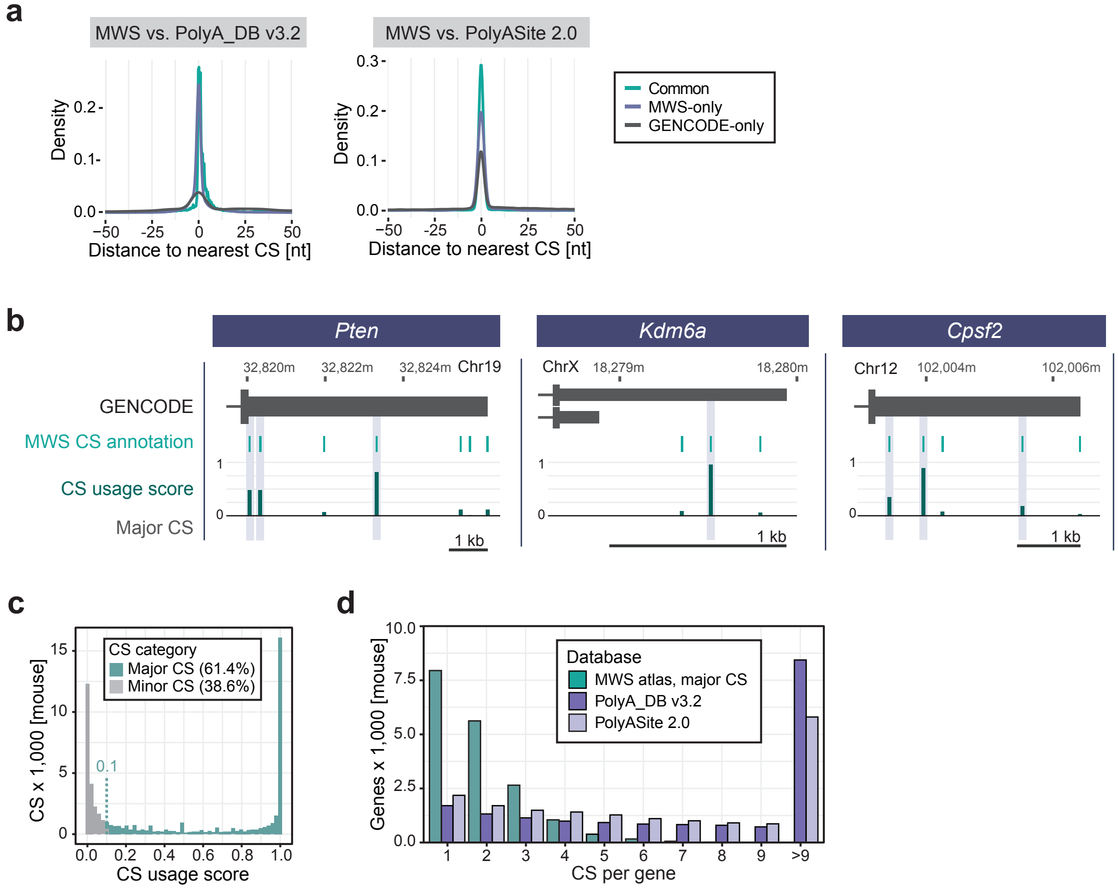

Supplementary Figure 2. Characterization of mRNA 3′ end CS in human and mouse primary cell types.

a Metaplots showing density of distances between CS in human MWS annotations and the nearest CS in two other CS databases. **b** GENCODE transcript annotations depicting the last exons of the mouse *Pten*, *Kdm6a*, and *Cpsf2* genes. Shown are chromosome coordinates (mm10) and the mouse MWS CS annotation, together with CS usage scores. Major CS are highlighted by the grey boxes. **c** CS usage score distribution for mouse MWS data. **d** Numbers of major MWS CS per gene compared to two other CS databases. Shown are all mouse protein coding genes.

Fansler, Mitschka & Mayr, Supplementary Figure 3

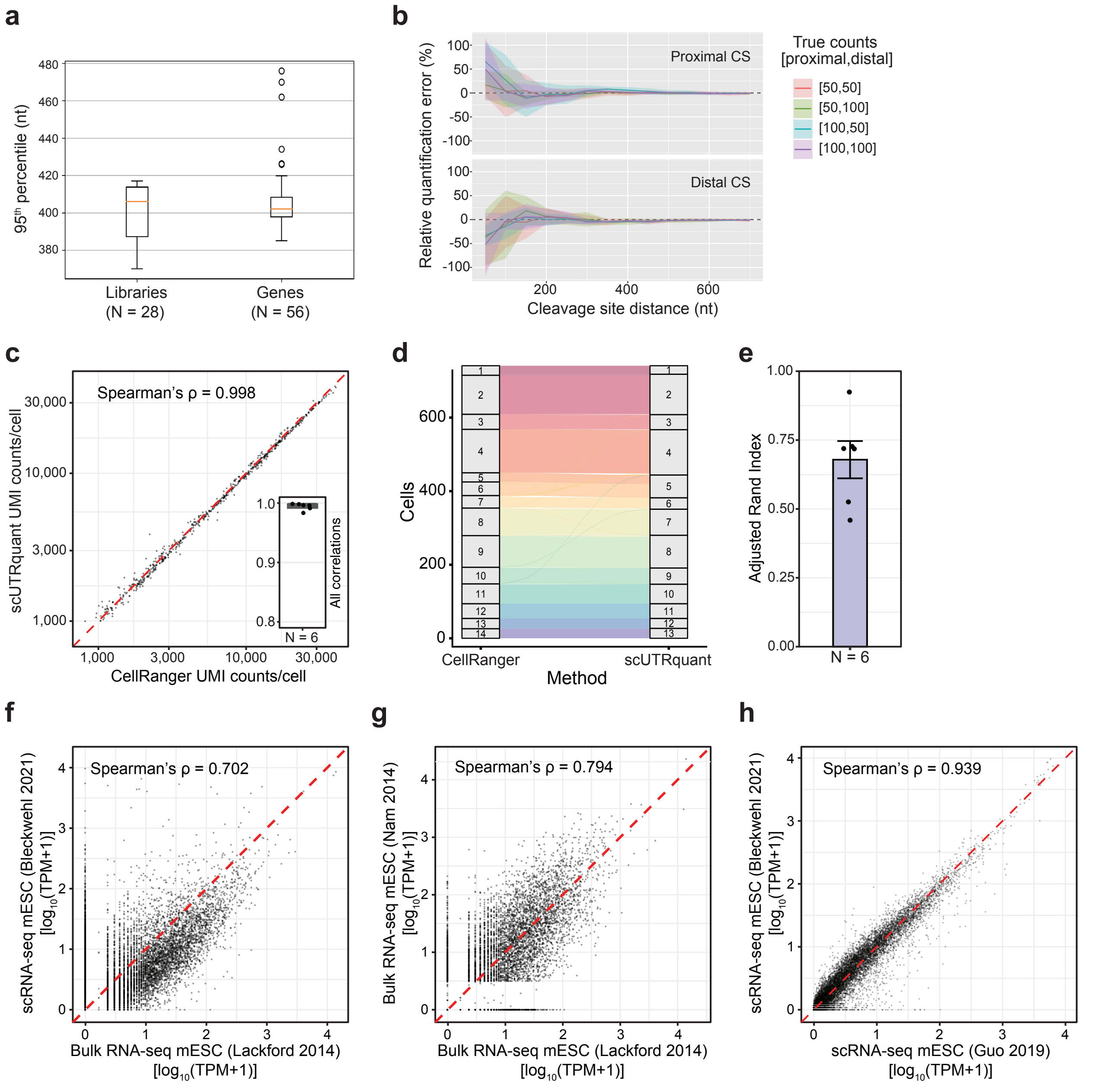

Supplementary Figure 3. scUTRquant-derived gene and 3′UTR expression is precise and accurate. **a** The cut-off for the UTRome truncation was empirically determined from Tabula Muris scRNA-seq data of 28 libraries and 56 curated genes. Box plot of distances to CS for the 95th percentile of reads in Tabula Muris scRNA-seq data. Box shows IQR with median and whiskers 1.5*IQR. **b** Error rates for transcript quantification with kallisto surveyed over a range of distances between CS. Lines indicate the mean of the relative errors from ten simulations for each of four simulated input read configurations. Ribbons indicate two standard deviations from the mean. Mean relative errors stabilize with a minimum CS distance of 200 nt. **c** Correlation of scUTRquant UMIs per cell obtained by scUTRquant and CellRanger for a mouse heart 10x Genomics demonstration dataset. Inset: Box shows IQR with median and whiskers 1.5*IQR for Spearman correlations of additional demostrations datasets. **d** Correspondence between clusters called from scUTR-quant and CellRanger gene counts for the dataset in (c). **e** Adjusted Rand Indexes for six mouse 10x Genomics demonstration sets comparing clusters called from UMI counts from CellRanger versus scUTR-quant. Mean and SE are shown. **f** Correlation of 3′UTR isoform counts obtained by bulk 3′ end sequencing and scUTRquant isoform counts of scRNA-seq of mouse ESCs. **g** Correlation of 3′UTR isoform counts between two experiments of bulk 3′ end sequencing for mouse ESCs. **h** Correlation of scUTRquant 3'UTR isoform counts between two experiments of scRNA-seq of mouse ESCs.

Fansler, Mitschka & Mayr, Supplementary Figure 4

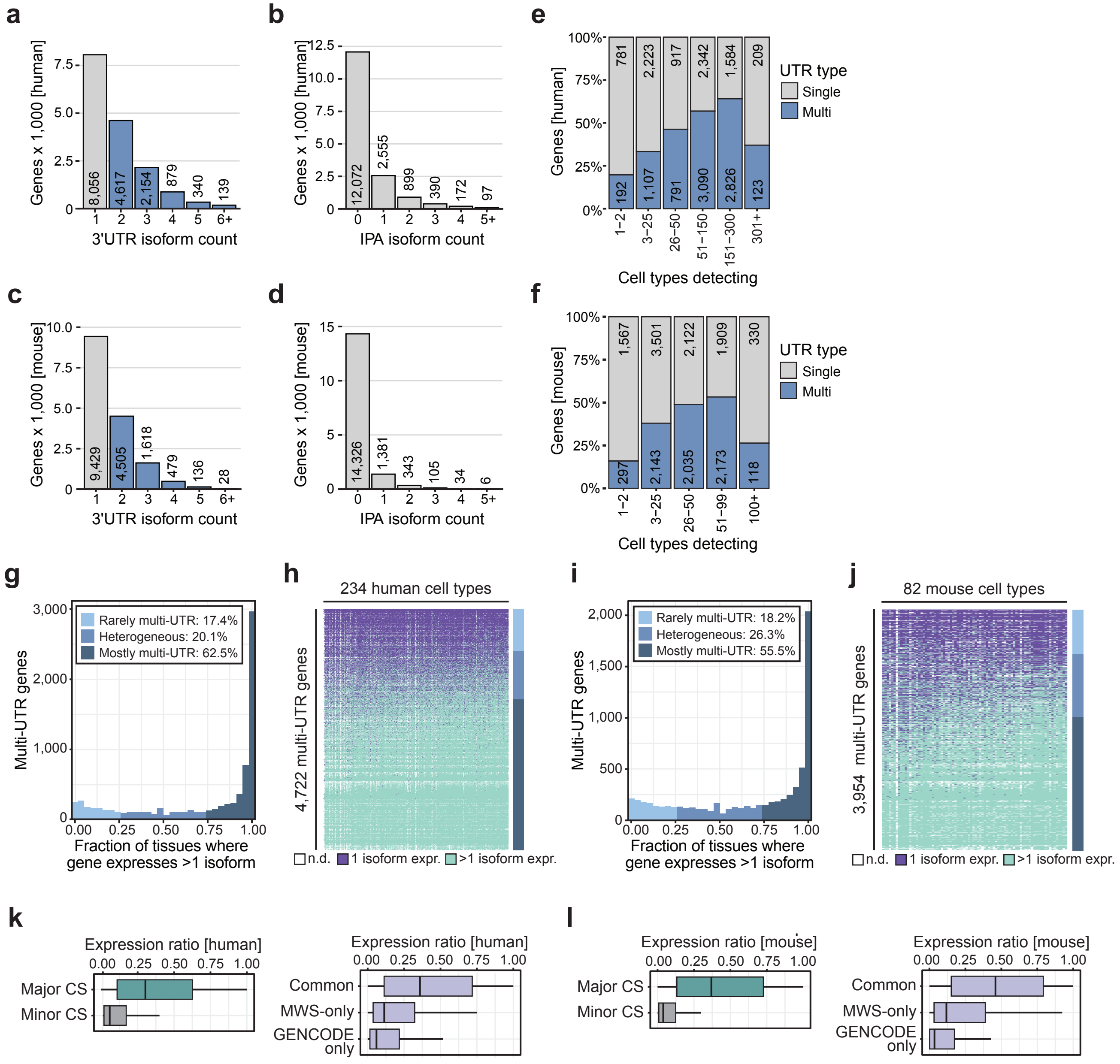

Supplementary Figure 4. Application of scUTRquant to 355 human and 119 mouse cell types for comprehensive identification of single- and multi-UTR genes and genes with IPA isoforms.
a Distribution of single- and multi-UTR genes when requiring a minimum expression of 10% for each alternative 3'UTR isoform located in the terminal exon from 355 cell types obtained from the Tabula Sapiens data set. **b** Same as (a), but for IPA events with a minimum expression of 10% for each IPA isoform in any cell type. **c** Same as (a), but classified from a survey of 119 mouse cell types. **d** Same as (b), but classified from a survey of 119 mouse cell types. **e** The fraction of single- and multi-UTR genes was plotted for human genes that are expressed broadly or in a cell type-restricted manner. Only genes detected in at least 50 cells were included. **f** Same as (e), but for mouse genes. **g** Histogram showing the frequency at which multi-UTR genes (gene TPM > 5) express more than one 3′UTR (relative expression >10%) across 234 human cell type samples. **h** Tile plot showing variability of 3′UTR isoform expression patterns in multi-UTR genes across 234 human cell types. n.d., not detetcted. **i** Same as (g) but with 82 mouse cell types. **j** Same as (h) but with 82 mouse cell types. **k** Fraction of gene expression contributed by individual 3′UTR isoforms resolved by CS category (left panel) or annotation category (right panel) of the associated CS. Analysis was performed for genes with total expression > 5 TPM across 234 human cell type samples. Only unmerged 3′UTR isoforms were included in the analysis. **l** Same as (k) but with 82 mouse cell types.

Fansler, Mitschka & Mayr, Supplementary Figure 5

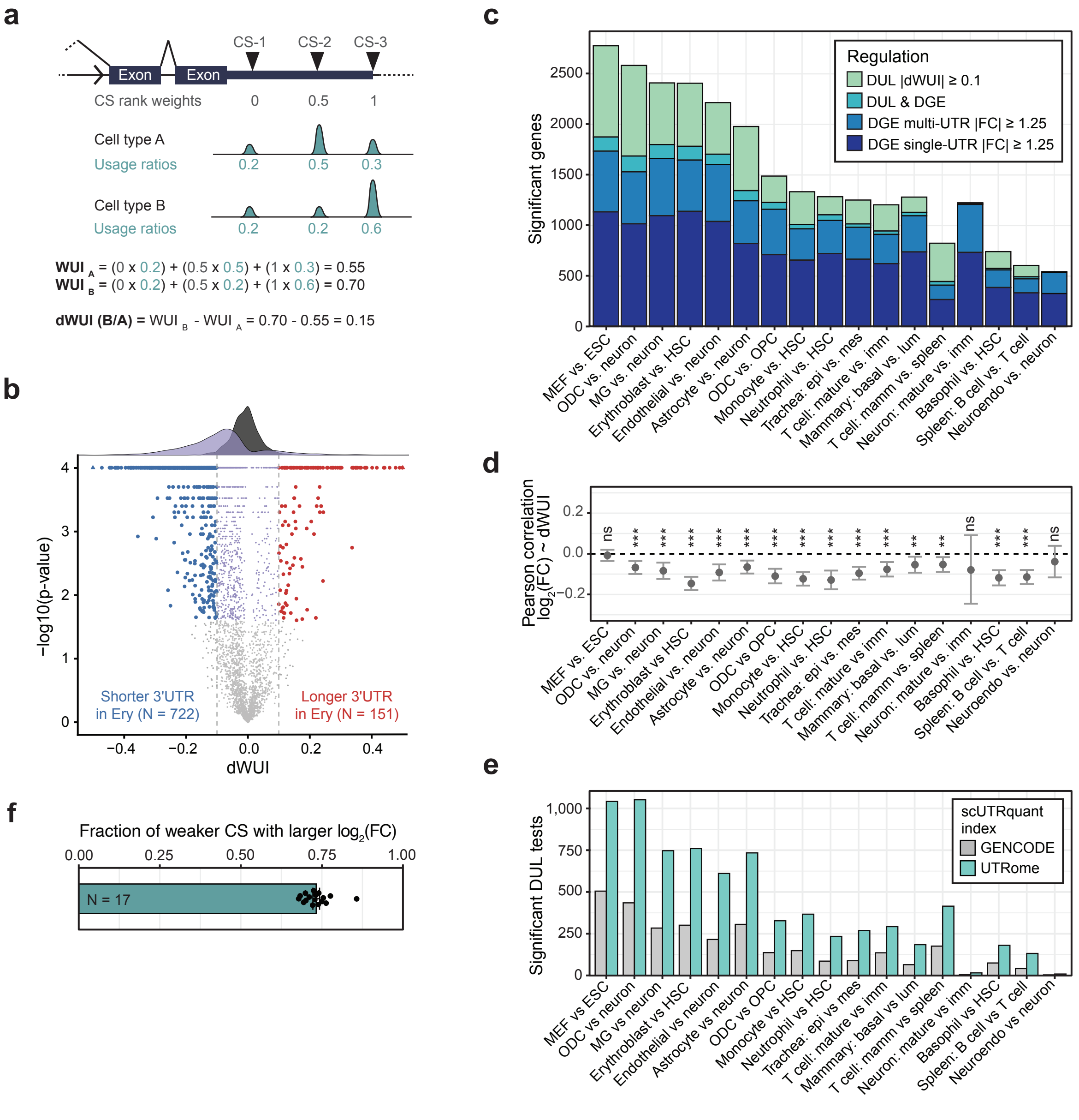

Supplementary Figure 5. Quantification of 3′UTR isoform expression changes across cell types.
a Example calculation of Weighted UTR expression Index (WUI) for two hypothetical cell types and 3′UTR isoform expression conditions. The 0.15 change in WUI (dWUI) across conditions indicates a relative shift to expression of longer isoforms. **b** Volcano plot showing p-values and the dWUI resulting from scUTRboot's WUI bootstrap test between hematopoetic stem cells (HSCs) and erythroblasts (Ery). Red, blue, significant dWUI (q-value < 0.05) and |dWUI| > 0.10. Purple, significant dWUI (q-value < 0.05), but with |dWUI| < 0.10 (N = 834); light grey, genes with non-significant dWUI (N = 1,693). **c** As in Fig. 3g, but applying a |FC|>1.25 cutoff for gene expression change. **d** Pearson's correlation coefficients for $\log_2$(FC) and WUI differences across 17 cell type comparisons shown in (c). Error bars indicate 95% confidence intervals and significance of correlations are indicated with ns (not significant) if p-value > 0.05, ** p < 0.01, *** p < 0.001. **e** As in Fig. 3h, but shown is the number of genes with significant DUL when two different CS annotations were used. The number of significant DUL tests is shown across 17 comparisons of mouse cell types using our UTRome compared with CS annotations from GENCODE. **f** For genes with two 3′UTR isoforms, the isoform with the larger fold change between cell types was calculated. Shown is the fraction of genes where the less abundant 3′UTR isoform showed a stronger fold change in the 17 cell type comparisons from Fig. 3g. Shown are mean and SE.

Fansler, Mitschka & Mayr, Supplementary Figure 6

Rank	Lengthening	Shortening	IPA increase	IPA decrease
1	FIP1L1	NUDT21	DBR1	RPL7A
2	RBBP6	CPSF6	SNRPD2	XRN1
3	RBM25	OGFOD1	NUDT21	CPSF2
4	RTF1	THOC2	DDX41	FIP1L1
5	SARS	THOC3	PAF1	CSTF1
6	SRSF7	THOC5	CPSF6	CPSF4
7	PCF11	THOC1	OGFOD1	CSTF3
8	PRPF40A	SEM1	PLRG1	TAF12
9	POT1	TCERG1	LSM5	UBA52
10	EXOSC5	GLE1	TTC27	CPSF3

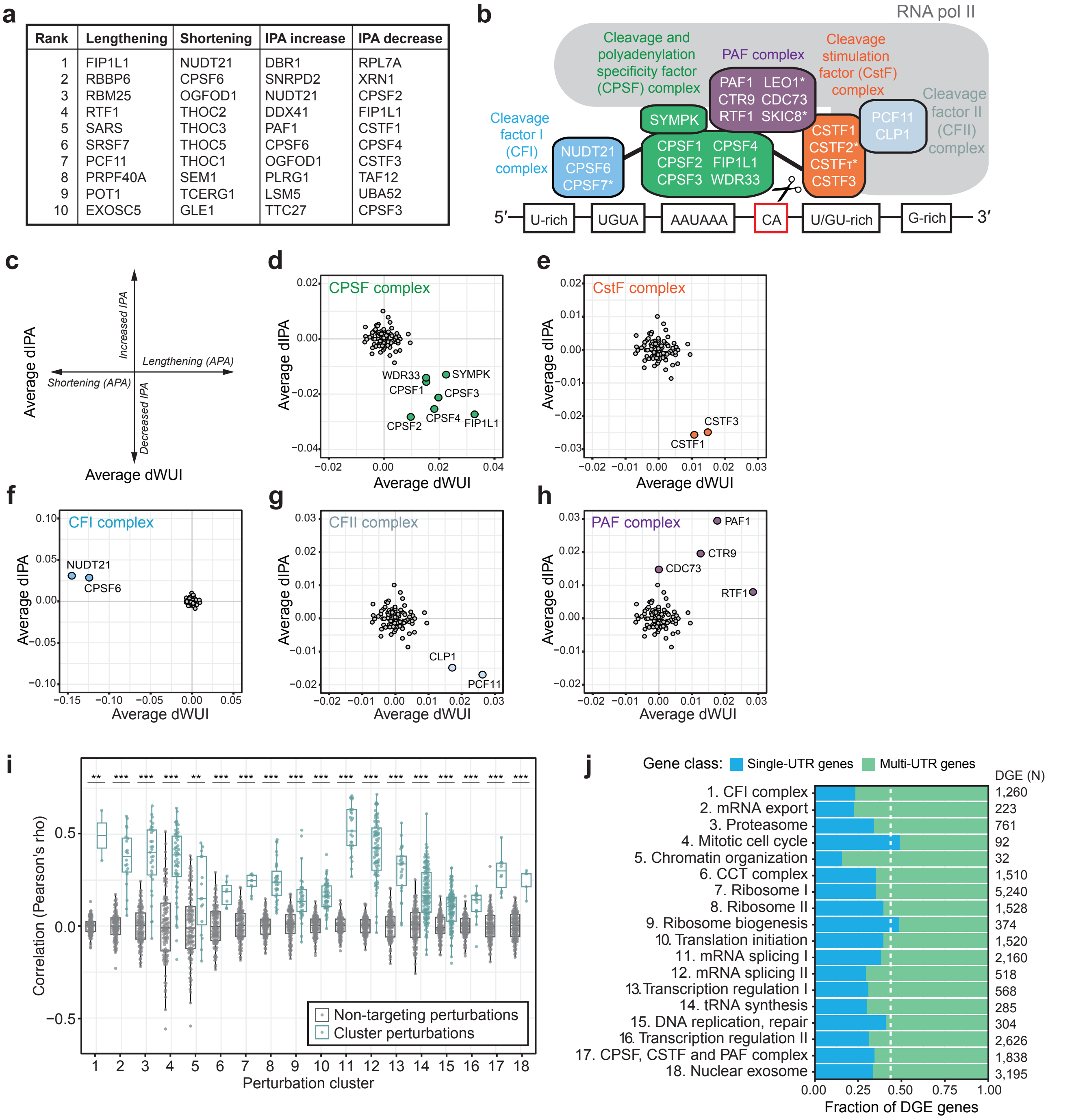

Supplementary Figure 6. scUTRquant identifies known APA and IPA regulators in a Perturb-Seq data set. **a** Ranked list of top ten regulators of APA and IPA among essential perturbations in K562 cells. A full list of all summarized perturbation effects is provided in Supplementary Data 6. **b** Schematic representation of the CPA machinery. Genes marked with asterisks are not present in the data set. **c** Plotting of global shifts in APA and IPA isoform expression patterns. **d-h** Knockdown effects of genes constituting the CPA machinery as shown in (b), and non-targeting control guide RNAs (grey, N = 97). Shown are: **d** CPSF complex (green), **e** CstF complex (orange), **f** CFI complex (blue), **g** CFII complex (light blue) and **h** PAF complex (purple). **i** Plot showing the Pearson correlations between the average z-scaled dWUIs from K562 clusters and z-scaled dWUIs from individual perturbations in RPE1 cells. Boxes show IQR with median and whiskers 1.5*IQR. Statistical significance was calculated using a one-sided Mann-Whitney test (** q-value < 0.01, *** q-value < 0.001) for targeted perturbations (blue) having higher correlation than non-targeting perturbations (black). **j** Fraction of single- and multi-UTR genes among all DGE genes with TPM > 5, |FC| > 1.5 and q < 0.05 (see Fig. 4c) for each cluster. The white dashed line indicates the ratio of single- to multi-UTR genes among all analyzed genes. The number of genes with DGE in each cluster is shown on the right.

Fansler, Mitschka & Mayr, Supplementary Figure 7

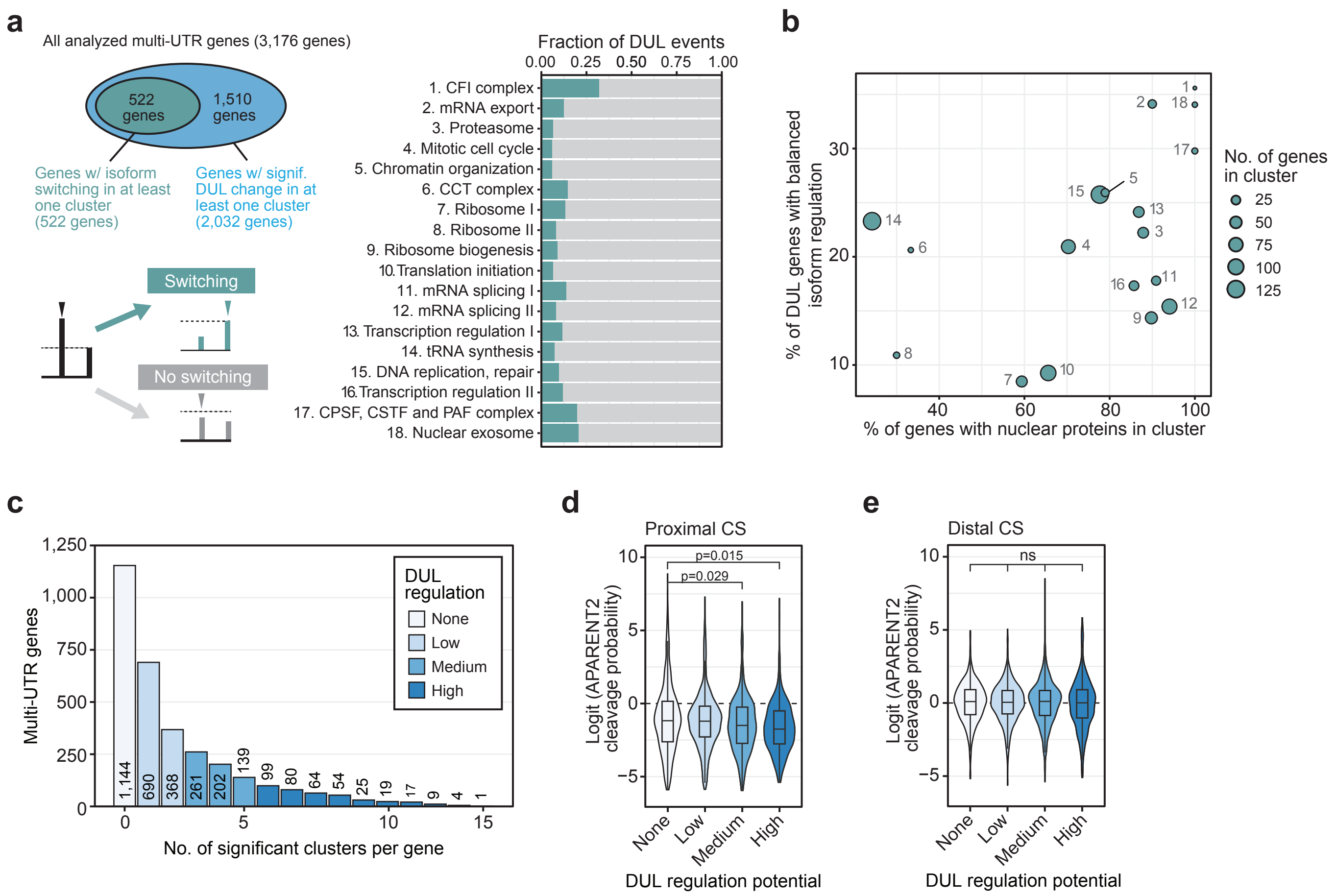

Supplementary Figure 7. The regulatory potential with respect to APA is mostly determined by the proximal CS.

a Overview of multi-UTR genes undergoing DUL changes in any of the 18 clusters with or without switching the dominantly expressed isoform relative to the non-targeting condition. Right panel: Fraction of isoform switching events in DUL genes resolved by individual clusters. **b** Dot plot showing the relationship of nuclear protein localization of APA regulators and coordinated isoform expression changes among DUL genes per cluster. Cluster numbers as in (a). **c** Histogram of multi-UTR genes showing the number of perturbation clusters that cause significant DUL changes. Genes were categorized into one of four groups based on their potential for dynamic regulation. **d** Analysis of predicted CS efficiencies across gene groups exhibiting different regulatory potential for proximal CS. Logit-transformed APARENT2 cleavage probabilities are used to estimate CS strength. Box shows IQR with median and whiskers 1.5*IQR. Statistical significance was calculated using a two-sided Mann-Whitney test. **e** As in (d), but shown are logit-transformed APARENT2 cleavage probabilities for distal CS (n.s. not significant).

Fansler, Mitschka & Mayr, Supplementary Figure 8

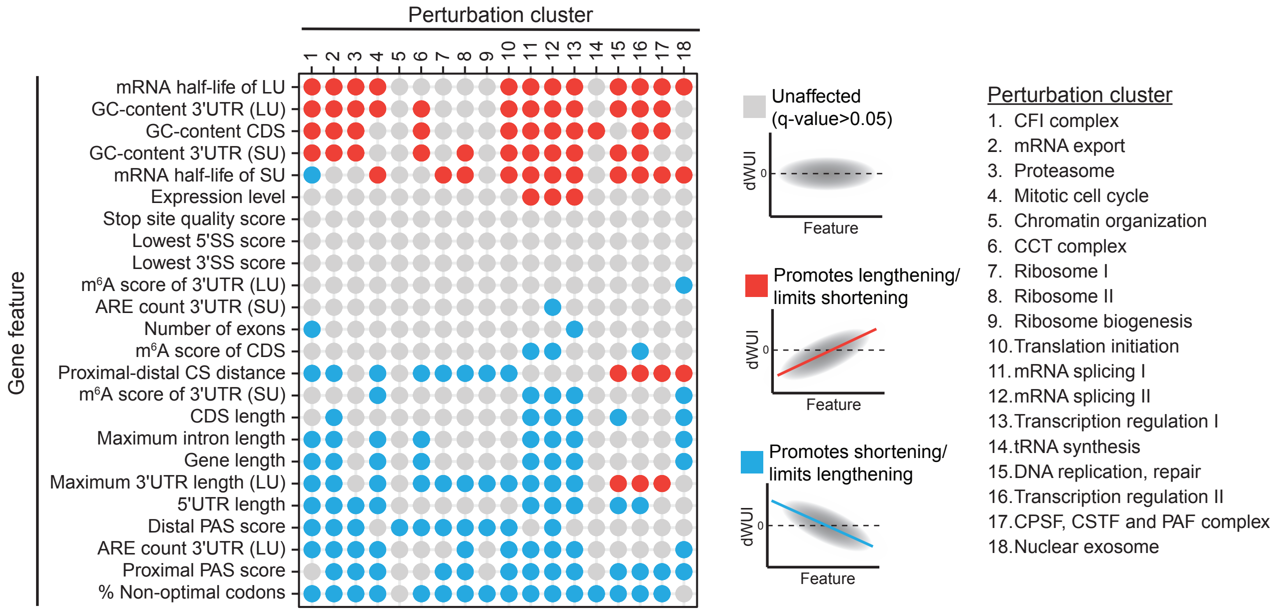

Supplementary Figure 8. Correlation anaylsis of cluster-specifc 3'UTR isoform regulation and gene features.

Gene and mRNA features that correlate with changes in 3′UTR isoform expression. Summarized results of correlations between each feature and each perturbation cluster. Grey dots represent correlations with FDR-corrected p-values > 0.05, while red and blue dots mark significant positive or negative correlations between features and average dWUI values in each perturbation cluster with FDR-corrected p-values <0.05. ARE, AU-rich element; PAS polyadenylation signal; SS, splice site.
