# Supplementary material for: Quantifying 3′UTR length from scRNA-seq data reveals changes independent of gene expression
Source: Nat Commun. 2024 May 14;15:4050. doi: 10.1038/s41467-024-48254-9 (PMC11094166; doi:10.1038/s41467-024-48254-9)
Supplement: Supplementary file 12 — Reporting Summary [file 41467_2024_48254_MOESM12_ESM.pdf]

nature portfolio

Corresponding author(s): Christine Mayr

Last updated by author(s): Apr 3, 2024

Reporting Summary

Nature Portfolio wishes to improve the reproducibility of the work that we publish. This form provides structure for consistency and transparency in reporting. For further information on Nature Portfolio policies, see our Editorial Policies and the Editorial Policy Checklist.

Statistics

For all statistical analyses, confirm that the following items are present in the figure legend, table legend, main text, or Methods section.

n/a	Confirmed	
☐	☒	The exact sample size (*n*) for each experimental group/condition, given as a discrete number and unit of measurement
☐	☒	A statement on whether measurements were taken from distinct samples or whether the same sample was measured repeatedly
☐	☒	The statistical test(s) used AND whether they are one- or two-sided<br>*Only common tests should be described solely by name; describe more complex techniques in the Methods section.*
☐	☒	A description of all covariates tested
☐	☒	A description of any assumptions or corrections, such as tests of normality and adjustment for multiple comparisons
☐	☒	A full description of the statistical parameters including central tendency (e.g. means) or other basic estimates (e.g. regression coefficient) AND variation (e.g. standard deviation) or associated estimates of uncertainty (e.g. confidence intervals)
☐	☒	For null hypothesis testing, the test statistic (e.g. *F*, *t*, *r*) with confidence intervals, effect sizes, degrees of freedom and *P* value noted<br>*Give P values as exact values whenever suitable.*
☒	☐	For Bayesian analysis, information on the choice of priors and Markov chain Monte Carlo settings
☒	☐	For hierarchical and complex designs, identification of the appropriate level for tests and full reporting of outcomes
☐	☒	Estimates of effect sizes (e.g. Cohen's *d*, Pearson's *r*), indicating how they were calculated

Our web collection on statistics for biologists contains articles on many of the points above.

Software and code

Policy information about availability of computer code

Data collection	No software for data collection was used.
Data analysis	The following open source software was used in this study:<br>APARENT-ResNet v1.0.2; aricode v1.0.2; BEDTools v2.30; bustools v0.40.0; cleanUpdTSeq v1.32; cutadapt v3.5; Cytoscape v3.9.1; GeneOverlap v1.32.0; GenomicScores v2.10.0; ggplot2 v3.4.1; GNU datamash v1.7; g:Profiler ve109_eg56_p17_773ec798; HISAT2 v2.2.1; htslib v1.14; pandas v1.3; PEAR v0.9.6; Python v3.9; R v4.2.2; samtools v1.14; scran v1.26.0; SingleCellExperiment v1.20.0; slamdunk v0.4.3; snakemake v6.8; sra-tools v2.11; stringApp v2.0.1; umi_tools v1.1.2.<br>All original code has been made publicly available on Github as of the date of publication. The repositories for processing pipelines are listed below and in the corresponding Methods sections:<br>The code to generate the human MWS annotation is deposited at https://github.com/Mayrlab/hcl-utrome (https://doi.org/10.5281/zenodo.8118411). The code to generate the mouse MWS annotation is deposited at https://github.com/Mayrlab/mca-utrome (https://doi.org/10.5281/zenodo.8118415). The code to process bulk HSPC data with the mouse MWS annotation is deposited at https://github.com/Mayrlab/sommerkamp20 (https://doi.org/10.5281/zenodo.10892209). The code to process SLAM-seq data and compute isoform-specific mRNA half-lives is deposited at https://github.com/Mayrlab/slam-k562-utrome (https://doi.org/10.5281/zenodo.10887801). The code to generate custom truncated transcriptomes for the scUTRquant pipeline is deposited at https://github.com/Mayrlab/txcutr-db (https://doi.org/10.5281/zenodo.8118404).<br>The repositories for original software intended for reuse are listed below and in the corresponding Methods sections:<br>The scUTRquant pipeline is deposited at https://github.com/Mayrlab/scUTRquant (https://doi.org/10.5281/zenodo.8118393). The source code for the customized version of kallisto is deposited at https://github.com/mfansler/kallisto/releases/tag/v0.46.2sq (https://doi.org/10.5281/zenodo.10902020). The scUTRboot R package is deposited at https://github.com/mfansler/scutrboot (https://doi.org/10.5281/zenodo.8057843). The txcutr Bioconductor package is deposited at https://bioconductor.org/packages/txcutr (https://

doi.org/10.18129/B9.bioc.txcutr). The codonopt R package for computing codon optimality is available at https://github.com/mfansler/codonopt (https://doi.org/10.5281/zenodo.10845962).
The repositories for analyses and figures presented in the manuscript are listed below:
The code to characterize the human MWS annotation is deposited at https://github.com/Mayrlab/hcl-analysis (https://doi.org/10.5281/zenodo.10892181). The code to characterize the mouse MWS annotation is deposited at https://github.com/Mayrlab/mca-analysis (https://doi.org/10.5281/zenodo.10892185). The code to characterize peak widths in Tabula Muris data is deposited at https://github.com/Mayrlab/tmuris-peaks (https://doi.org/10.5281/zenodo.10895190). The code to characterize kallisto resolution for overlapping transcripts is deposited at https://github.com/Mayrlab/kallisto-overlap (https://doi.org/10.5281/zenodo.10895237). The code to characterize isoform expression across the Tabula Sapiens dataset is deposited at https://github.com/Mayrlab/atlas-hs (https://doi.org/10.5281/zenodo.10895336). The code to characterize isoform expression across mouse datasets is deposited at https://github.com/Mayrlab/atlas-mm (https://doi.org/10.5281/zenodo.10895351). The code to process and analyze the Perturb-seq data set is deposited at https://github.com/Mayrlab/gwps-sq (https://doi.org/10.5281/zenodo.10895730). Additional code to generate analyses and figures is deposited at https://github.com/Mayrlab/scUTRquant-figures (https://doi.org/10.5281/zenodo.10910013).

For manuscripts utilizing custom algorithms or software that are central to the research but not yet described in published literature, software must be made available to editors and reviewers. We strongly encourage code deposition in a community repository (e.g. GitHub). See the Nature Portfolio guidelines for submitting code & software for further information.

Data

Policy information about availability of data

All manuscripts must include a data availability statement. This statement should provide the following information, where applicable:

- Accession codes, unique identifiers, or web links for publicly available datasets
- A description of any restrictions on data availability
- For clinical datasets or third party data, please ensure that the statement adheres to our policy

This paper analyzes existing, publicly available data. The accession numbers for the datasets are listed below and in the corresponding Methods sections discussing their processing.
The Mouse Cell Atlas v1.136 data used in this study are available in the GEO database under accession code GSE108097 [https://www.ncbi.nlm.nih.gov/geo/query/acc.cgi?acc=GSE108097].
The Human Cell Landscape37 data used in this study are available in the GEO database under accession code GSE134355 [https://www.ncbi.nlm.nih.gov/geo/query/acc.cgi?acc=GSE134355].
The bulk 3′-seq FACS-sorted HSCs data used in this study are available in the ArrayExpress database under the accession code E-MTAB-7391 [https://www.ebi.ac.uk/biostudies/arrayexpress/studies/E-MTAB-7391].
The scRNA-seq mouse HSPC data used in this study are available in the GEO database under the accession code GSE107727 [https://www.ncbi.nlm.nih.gov/geo/query/acc.cgi?acc=GSE107727].
The mESC scRNA-seq data used in this study are available in the GEO database under the accession codes GSM3629847 [https://www.ncbi.nlm.nih.gov/geo/query/acc.cgi?acc=GSM3629847], GSM3629848 [https://www.ncbi.nlm.nih.gov/geo/query/acc.cgi?acc=GSM3629848], and GSM4694997 [https://www.ncbi.nlm.nih.gov/geo/query/acc.cgi?acc=GSM4694997].
The mouse brain data used in this study are available in the GEO database under the accession code GSE129788 [https://www.ncbi.nlm.nih.gov/geo/query/acc.cgi?acc=GSE129788].
The Tabula Muris data used in this study are available in the GEO database under the accession code GSE109774 [https://www.ncbi.nlm.nih.gov/geo/query/acc.cgi?acc=GSE109774].
The raw Tabula Sapiens data used in this study is on AWS under restricted access due to data privacy restrictions; access can be requested at https://tabula-sapiens-portal.ds.czbiohub.org/whereisthedata.
The K562 6-day essential gene Perturb-seq experiments58 used in this study are available in the SRA database under accession codes SRR19653800-SRR19653847 [https://www.ncbi.nlm.nih.gov/Traces/study/?acc=SAMN28561243].
The RPE1 RPE1 7-day essential gene Perturb-seq experiments58 used in this study are available in the SRA database under accession codes SRR19653359-SRR19653414 [https://www.ncbi.nlm.nih.gov/Traces/study/?acc=SAMN28561244].
The cell annotations for the Perturb-seq experiments58 used in this study are available on figshare (https://doi.org/10.25452/figshare.plus.20029387.v1).
The m6A sites mapped by eTAM seq in HeLa data used in this study are available in the GEO database under accession code GSE211303 [https://www.ncbi.nlm.nih.gov/geo/query/acc.cgi?acc=GSE211303].
The K562 SLAM-seq data78 used in this study are available in the GEO database under accession code GSE126522 [https://www.ncbi.nlm.nih.gov/geo/query/acc.cgi?acc=GSE126522]. The codon stability coefficients data used in this study are from Figure 1-Source Data 278 available at https://doi.org/10.7554/eLife.45396.006.
The PolyASite 2.0 databases for human and mouse used in this study are available at https://polyasite.unibas.ch/atlas.
The PolyA_DB v3.2 database for human and mouse used in this study are available at https://exon.apps.wistar.org/polya_db/v3/misc/download.php.
The protein subcellular localization data used in this study are available in the Human Protein Atlas database (v22.0) at https://www.proteinatlas.org.
The STRING Database v11.5 data used in this study are available at https://version-11-5.string-db.org/.

Processed data generated in this study are available in the following locations:
The GTF and BED files for the MWS CS annotation in human and mouse data generated in this study are available on figshare (https://doi.org/10.6084/m9.figshare.23549526). The SingleCellExperiment and SummarizedExperiment objects generated in this study by scUTRquant and post-processing pipelines are available on figshare (https://doi.org/10.6084/m9.figshare.25513528). The intermediate data objects used to generate figures are available on figshare (https://doi.org/10.6084/m9.figshare.25529632).
The MWS CS annotations for human generated in this study are provided in Supplemental Data 1. The MWS CS annotations for mouse generated in this study are provided in Supplemental Data 2. The human gene annotations from analyzing 355 human cell types generated in this study are provided in Supplemental Data 3. The mouse gene annotations from analyzing 119 mouse cell types generated in this study are provided in Supplemental Data 4. The independence and correlation test results between significant DGE and DUL generated in this study are provided in Supplemental Data 5. The average DWUI and DIPA for K562 6-day essential perturbations generated in this study are provided in Supplemental Data 6. The APA regulator clusters and GO term analysis generated in this study are provided in Supplemental Data 7. The DGE and DUL significance testing results for APA regulator clusters generated in this study are provided in Supplemental Data 8. The correlation test results among DUL changes and genomic features generated in this study are provided in Supplemental Data 9.

Research involving human participants, their data, or biological material

Policy information about studies with human participants or human data. See also policy information about sex, gender (identity/presentation), and sexual orientation and race, ethnicity and racism.

Reporting on sex and gender	N/A
Reporting on race, ethnicity, or other socially relevant groupings	N/A
Population characteristics	N/A
Recruitment	N/A
Ethics oversight	N/A

Note that full information on the approval of the study protocol must also be provided in the manuscript.

Field-specific reporting

Please select the one below that is the best fit for your research. If you are not sure, read the appropriate sections before making your selection.

☒ Life sciences ☐ Behavioural & social sciences ☐ Ecological, evolutionary & environmental sciences

For a reference copy of the document with all sections, see nature.com/documents/nr-reporting-summary-flat.pdf

Life sciences study design

All studies must disclose on these points even when the disclosure is negative.

Sample size	For technical comparison with CellRanger counts, the available 10X Genomics demonstration data (N=6 mouse; N=3 human) were used. These were regarded as a technical standard and sufficient to characterize gene and UMI count correlations. We were limited by available datasets of bulk 3'-seq with corresponding scRNA-seq. The HSPC datasets provided N=4 bulk 3'-seq and N=3 scRNA-seq biological replicates, which we considered sufficient to characterize correlations both across and within the techniques. The mESC datasets provided N=2 bulk 3'-seq and N=2 scRNA-seq experimental replicates (across labs), which excluded drawing any statistical conclusions. The N=17 comparisons for DGE and DUL gene independence were selected to represent a diverse selection of differentiation endpoints.
Data exclusions	scUTRboot uses a heuristic minimum of 50 cells expressing a gene in both conditions (e.g., cell types) being compared, otherwise tests are omitted. This threshold for independent filtering was established prior to processing the data in this study. In primary tissue data, a minimum of 200 cells per cell type was used to exclude cell types that were too rare to reliably aggregate to pseudobulk. In the Perturb-seq data, a minimum cell count of 30 was used to exclude perturbations from clustering analysis.
Replication	To ensure reproducibility of bioinformatic processing and analyses, code for pipelines and analyses was version controlled and deposited in GitHub. All code was executed in Conda software environments and these environments were completely documented in pipelines and analyses, including both minimal environment definitions (YAML) for recreating environments across platforms (e.g., macOS, Linux) and complete environment definitions that captured the versions of all software installed in the environments. Pipelines used Snakemake to ensure the ability to rerun all pipeline steps with minimal setup. Pipelines start from sample sheets that direct downloading of raw data from persistent databases. Any additional, system-specific configuration parameters were documented in the pipeline repositories. Directions for requesting access to Tabula Sapiens data, the only controlled-access data processed in this study, were included in the Data Availability statement. Additionally, intermediate objects were deposited to Figshare (see Data Availability statement) to facilitate replication of downstream analysis code without the need for rerunning raw data processing pipelines. Analysis and figure generation code was executed in isolated RMarkdown documents that captured the versions of all software used in the code evaluation.
Randomization	Randomization was not relevant to this study. This study did not involve enrollment of patients or splitting of data into study arms.
Blinding	The selection of N=17 comparisons for DGE and DUL gene independence was done blindly, prior to DGE and DUL testing. Gene features used in APA regulation cluster correlation analysis were selected blindly.

Reporting for specific materials, systems and methods

We require information from authors about some types of materials, experimental systems and methods used in many studies. Here, indicate whether each material, system or method listed is relevant to your study. If you are not sure if a list item applies to your research, read the appropriate section before selecting a response.

Materials & experimental systems

n/a	Involved in the study
☒	☐ Antibodies
☒	☐ Eukaryotic cell lines
☒	☐ Palaeontology and archaeology
☒	☐ Animals and other organisms
☒	☐ Clinical data
☒	☐ Dual use research of concern
☒	☐ Plants

Methods

n/a	Involved in the study
☒	☐ ChIP-seq
☒	☐ Flow cytometry
☒	☐ MRI-based neuroimaging

Plants

Seed stocks	No plants were used in this study.
Novel plant genotypes	No plants were used in this study.
Authentication	No plants were used in this study.
